# Albuminuria and its associated biomedical factors among indigenous adults in Far North Queensland: a 7-year follow up study

**DOI:** 10.1186/s12882-015-0200-8

**Published:** 2015-12-10

**Authors:** Ming Li, Robyn McDermott

**Affiliations:** Centre for Population Health Research, Sansom Institute for Health Research, University of South Australia, IPC CWE-48, GPO Box 2471, Adelaide, SA 5001 Australia; Faculty of Medicine, Health & Molecular Sciences, James Cook University, Cairns, QLD 4870 Australia

**Keywords:** Albuminuria prevalence, Australian Indigenous populations, Overweight and obesity, Blood glucose and diabetes, Gamma-glutamyltransferase, Smoking

## Abstract

**Background:**

To document albuminuria prevalence and its associated factors in Aboriginal and Torres Strait Islander (TSI) adults with high renal and metabolic risks from 19 rural and remote north Queensland communities.

**Methods:**

One thousand nine hundred seventy-one indigenous adults were enrolled in 1998 and 566 completed follow up in 2007 in this population-based study. Measurements included weight, waist circumference (WC), blood pressure (BP), fasting glucose, lipids, gamma-glutamyltransferase (GGT), urinary albumin creatinine ratio (UACR), smoking, alcohol intake and physical activity (PA). Albuminuria was defined as an UACR > =2.5 g/mol in males and > =3.5 g/mol in females. The association between albuminuria and biomedical factors was assessed with generalised linear modelling.

**Results:**

Baseline albuminuria prevalence was 19.7 % (95 % CI: 18.0–21.6 %). Follow up prevalence was 42.4 % (95 % CI: 38.4–46.5 %) among the 566 adults having the 2^nd^ UACR measurements. Follow-up albuminuria was associated with fasting glucose of 5.4 mmol/L (OR 2.5, 95 % CI 1.5–4.2), GGT tertiles in a dose-response manner (OR 2.0 for 2^nd^ and 3.7 for 3^rd^ tertile, p for trend <0.001), and abdominal overweight and obesity (OR 2.1, 95 % CI 1.1–3.9 and 5.4, 95 % CI: 2.2–13.5 respectively). Aboriginal people with diabetes were three times more likely of having albuminuria compared to TSI counterparts, while TSI smokers had twice the likelihood (95 % CI 1.2–3.2). At both baseline and follow up, albuminuria was more prevalent among older participants.

**Conclusions:**

Indigenous Australians in north Queensland are at high risk of albuminuria. Overweight and obesity, glycaemia, increased GGT, and smoking were associated with albuminuria at baseline and/or follow up.

**Electronic supplementary material:**

The online version of this article (doi:10.1186/s12882-015-0200-8) contains supplementary material, which is available to authorized users.

## Background

Albuminuria is a pathological condition with excessive leakage of protein into the urine. It is an indicator of kidney damage and is an early manifestation of impaired kidney function leading to chronic kidney disease (CKD). CKD is becoming a worldwide public health problem [1] where individuals with kidney failure require dialysis or transplantation that poses a substantial pressure on the health-care system [2]. With an aging population and increasing prevalence of diabetes and other chronic diseases, early detection and appropriate management of CKD becomes important [3]. From a public health perspective, screening for albuminuria to prevent CKD and cardiovascular disease (CVD) is feasible and cost effective in high risk populations [4–7], based on the consistent results among large population studies that albuminuria and proteinuria strongly and independently predict the risk of CKD progress, CVD, and all cause-mortality [8–10].

The Australian Diabetes, Obesity and lifestyle Study (AusDiab) reported a prevalence of micro albuminuria and macro albuminuria, defined as UACR > =2.5 g/mol for males and > =3.5 g/mol for females in a spot urine test, among over 25 year old Australians in 2000 as 6.0 % and 0.6 % respectively [11]. A five-year follow up of the same population showed a 0.8 % annual incidence of albuminuria [12]. Aboriginal and TSI people are at substantially greater risk of having poorer health outcomes including CKD and associated hospitalization and deaths compared to non-Indigenous Australians [13]. Among screened volunteers from an isolated Northern Territory (NT) Aboriginal community during 1992–1995, 26 % of adults had micro albuminuria and 24 % had overt albuminuria [14], similar to the rate from screening surveys conducted in Aboriginal communities in South Australia (SA), Western Australia (WA) and Queensland (QLD) during 1995–1997 [15, 16]. We have shown that albuminuria predicts incidence of diabetes, hypertension, and coronary heart diseases among Indigenous adults in Far North Queensland [17–20]. Therefore, understanding albuminuria and its predictors is important in preventing and managing effectively these morbidities. However, the associated factors of albuminuria among Indigenous Australians are not well reported, and to date CKD has largely been focused on the evaluation of end-stage kidney disease (ESKD) using clinical data [13].

Here we aim to document the prevalence of albuminuria and its associated factors in two Indigenous populations from north Queensland.

## Methods

### Study population

This study included a subgroup of 1971 Indigenous participants aged over 18 years in far north Queensland in the “Well Person’s Health Check” (WPHC). WPHC was a population based wellness screening program in 19 rural and remote Indigenous communities across three health districts during 1998–2000. Methods for this cross sectional study have been reported in detail elsewhere [21]. Briefly, all Indigenous residents of the communities aged 13 years and over were invited to attend a health check via local health services, community councils, and community groups. Based on the local census data, the study achieved a participation rate of 44.5 % with greater participation noted in smaller communities. The follow-up data were collected during 2004–2007. Participants overall were not different demographically from the age and sex distribution of the Australian Indigenous population as a whole. Written informed consent was obtained from participants. The study protocols were approved by the Cairns Base Hospital Human Research Ethics Committee with support from the peak Indigenous Health Organizations, Apunipima Cape York Health Council and the Torres Strait and Northern Peninsula Area Health Council.

### Measurements

Urine specimens provided by participants in sterile 50 mL containers were from the first morning void or a sample at least two hours from the most recent void. Dipstick urinalysis (Combur-test, Roche) tested the samples for protein, pH, nitrites, leucocytes and blood. UACR was measured by immunoassay in g/mol in Cairns Base Hospital. Albuminuria was defined as having UACR > =2.5 g/mol in males and > =3.5 g/mol in females based on a single urine test.

Participants in light clothes were weighed to the nearest 0.1 kg. Height and WC were recorded to the nearest centimetre with the latter measured by the same technician at the level of the umbilicus. BP was the average of three measurements taken sitting after 10 min rest using a Dinamap model 800 automated blood pressure monitor (Critikon; Tampa FL, US). PA was self-reported and was categorized using the WHO criteria in which ‘enough’ means doing moderate to vigorous physical activity for more than 30 min/day for 5 days in the week before the survey. Daily number of cigarettes smoked and alcohol intake was also self-reported.

GGT, fasting total cholesterol, high density lipoprotein cholesterol (HDLC), triglycerides, and glucose were measured on fasting blood. GGT was measured using the kinetic photometric procedure with Cobas Integra 800 (Roche Diagnostics, www.roche-diagnostics.us/). Blood lipids and blood glucose were measured using photometric enzyme endpoint assay with Cobas Integra 700/400 (Roche Diagnostics, www.roche-diagnostics.us/).

Abdominal overweight was defined using WHO criteria as WC greater than 80 cm in females and 94 cm in males and obesity as greater than 88 cm in female and 102 in male. Baseline hypertension was ascertained either by detection of high BP at examination (measured BP > =140/90 (mmHg) or previous confirmed diagnosis or currently prescribed antihypertensive medication (by medical record review). Dyslipidaemia was defined as having triglycerides > =2.0 mmol/L or HDLC <1.0 mmol/L recommended by the National Heart Foundation. Diabetes was defined as either clinical diagnosis verified by the participants’ medical records, a 2 h oral glucose tolerance test, or fasting blood glucose level >7.0 mmol/L.

### Analysis

Prevalence of albuminuria at baseline and follow up was estimated using UACR measurements at two time points respectively, and its 95 % confidence interval was computed using the binomial distribution. Prevalence was compared by baseline characteristics including age group (<35 years vs > =35 years), sex, ethnicity, hypertension, fasting glucose or diabetes, blood lipids, GGT categories, smoking and drinking habits. The association between albuminuria at follow up and biomedical factors were explored using Generalised Linear Model with family of “binomial” and link of “logit”. Prevalence rate ratio (OR) was adjusted for age, sex and ethnicity. The analysis was carried out using STATA 13 (STATAcorp, College Station, Texas, USA) and significance level was set at two-sided P < 0.05.

## Results

Among 1971 Indigenous participants aged 18 years and over (mean age 38.7 years) with available UACR measurements in the 19 communities, 64 % were overweight/obese. The prevalence of hypertension was 31 % and diabetes was 17 %. 58 % reported smoking and 70 % self-reported as alcohol drinkers. 389 had albuminuria at baseline (19.7 %, 95 % CI: 18.0–21.6 %). Those with albuminuria were more likely to be older, TSIs but not different by sex (Table 1). Overweight/obesity, and higher BP, glucose, and GGT were associated with baseline albuminuria independent of age, sex and ethnicity.

**Table 1:** Baseline characteristics of Indigenous adults in 19 communities from North Queensland 1998–2000 by albuminuria at baseline

	Albuminuria No = 1582	Albuminuria Yes = 389	Total N = 1971
	Mean or % (95% CI)	Mean or % (95% CI)	Mean or % (95% CI)
Age (Years) *	36.8 (36.1-37.5)	46.5 (45.0-48.0)	38.7 (38.1-39.4)
Female %	50.6 (48.2-53.1)	48.8 (43.9-53.8)	50.3 (48.1-52.5)
Aboriginal % *	54.0 (51.5-56.4)	43.4 (38.6-48.4)	51.9 (49.7-54.1)
WC (CM) *	95.8 (95.0-96.6)	104.4 (102.8-106.0)	97.5 (96.8-98.2)
Abdominal overweight and obesity * %			
Overweight	19.6 (17.5-21.8)	19.0 (14.8-23.9)	19.5 (17.6-21.5)
Obesity	41.4 (38.8-44.1)	58.3 (52.5-63.8)	44.4 (42.0-46.8)
BMI (kg/m^2^) *	27.8 (27.5-28.1)	30.4 (29.6-31.1)	28.3 (28.0-28.6)
BMI % *			
25-29.9	27.0 (24.9-29.3)	25.2 (21.1-29.9)	26.7 (24.8-28.7)
> = 30	34.4 (32.1-36.8)	50.9 (45.9-55.9)	37.7 (35.5-39.8)
SBP (mmHg) *	129.2 (128.3-130.1)	140.8 (138.7-142.8)	131.5 (130.6-132.3)
DBP (mmHg) *	71.0 (70.3-71.7)	77.5 (76.2-78.8)	72.3 (71.7-72.9)
Hypertension % *	26.4 (24.3-28.6)	49.9 (44.9-54.8)	31.0 (29.0-33.1)
Glucose (mmol/L) *	5.5 (5.4-5.7)	7.0 (6.7-7.4)	5.8 (5.7-6.0)
Diabetes % *	12.4 (10.9-14.1)	35.2 (30.6-40.1)	16.9 (15.3-18.6)
Cholesterol (mmol/L) *	4.97 (4.92-5.02)	5.13 (5.02-5.24)	5.00 (4.96-5.05)
Triglycerides (mmol/L) *	1.87 (1.79-1.94)	2.12 (1.95-2.29)	1.92 (1.85-1.99)
HDL (mmol/L)	1.12 (1.11-1.14)	1.18 (1.15-1.20)	1.13 (1.12-1.15)
Dyslipidaemia %	46.8 (44.3-49.3)	49.5 (44.4-54.5)	47.3 (45.1-49.5)
GGT (IU) *	50.2 (47.0-53.4)	57.5 (51.6-63.3)	51.6 (48.8-54.5)
GGT > 50 % *	28.7 (26.5-31.0)	38.2 (33.4-43.2)	30.6 (28.5-32.7)
Smoker % *	60.4 (58.0-62.8)	49.2 (44.3-54.2)	58.2 (56.0-60.4)
Drinking status %			
Moderate	23.4 (21.4-25.6)	20.4 (16.6-24.7)	22.8 (21.0-24.7)
Risky	49.3 (46.8-51.8)	46.8 (41.8-51.9)	48.8 (46.6-51.1)
PA sufficient %	25.6 (23.5-27.8)	18.8 (15.2-23.0)	24.3 (22.4-26.2)

*P < 0.05 from ttest or chi square test or corresponding non-parametric tests

Abdominal overweight was defined using WHO criteria as WC greater than 80 cm in females and 94 cm in males and obesity as greater than 88 cm in female and 102 in male

Hypertension was ascertained either by detection of high BP at examination (measured BP > =140/90 (mmHg) or previous confirmed diagnosis or currently prescribed antihypertensive medication (by medical record review)

Diabetes was defined as either clinical diagnosis verified by the participants’ medical records or a 2 hour oral glucose tolerance test, or fasting blood glucose level >7.0 mmol/L

Dyslipidaemia was defined as having triglycerides > =2.0 mmol/L or HDLC <1.0 mmol/L recommended by National Heart Foundation

**Fig. 1:**
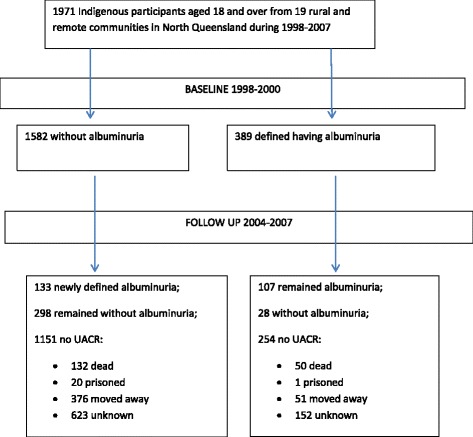
UACR measurements and albuminuria among the participants at baseline and follow up surveys during 1998–2007

Figure 1 showed participants during the follow up period (1998–2007) by baseline albuminuria categories. In total, 566 people had the 2^nd^ UACR measurements, 182 had died, 427 had moved away from the communities, 21 were in prison, and 775 left with no reason recorded. The baseline characteristics by follow up status are summarized in the Additional file 1: Table S1. Briefly, compared to those who completed follow up, those not followed up were significantly younger, more likely to be Aboriginal with significantly healthier biomedical measurements including BMI, systolic BP, and fasting blood glucose, but were also more likely to smoke tobacco, and/or drink alcohol. Among participants with baseline albuminuria, more had died or did not complete follow up, and fewer were imprisoned or moved away from the communities. People having the 2^nd^ UACR test were evenly distributed by baseline albuminuria categories.

Among the complete cohort, 201 were Aboriginal, 303 were TSI people and 62 were joint descendants. Aboriginal people had higher diastolic BP, triglycerides, and GGT, and a higher percentage of risky drinkers, but lower WC, BMI, and fasting glucose than the TSI participants. The demographic and biomedical characteristics of the complete cohort were similar to those among baseline population (Additional file 2: Table S2).

**Fig. 2:**
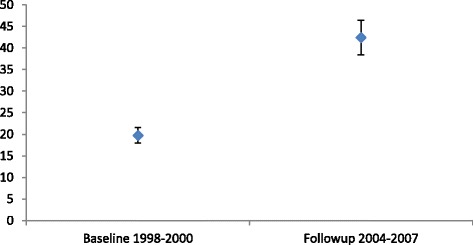
Albuminuria prevalence among Indigenous adults in 19 rural and remote communities in North Queensland 1998–2007

At the follow up, 133 new cases of albuminuria developed among those with normal UACR at baseline, while 107 baseline cases remained as albuminuria. Twenty-eight went normal at the follow up (Fig. 1). The mean UACR of the 28 cases (12 of whom were females) was 1.2 g/mol at follow up. To sum up, a total of 240 albuminuria cases were identified among 566 participants giving an overall follow up prevalence of albuminuria 42.4 % (95 % CI: 38.8–46.5 %). The prevalence of albuminuria was higher than the baseline as shown in Fig. 2.

Table 2 shows the biomedical and behavioral characteristics of the complete cohort by follow up albuminuria. Participants with albuminuria at follow up were older or had higher BMI, BP, cholesterol, triglycerides, and GGT. Follow up albuminuria was not associated with drinking and PA and it was not significantly different by sex and ethnicity.

**Table 2:** Baseline characteristics of Indigenous adults in 19 communities from North Queensland by albuminuria at follow up

	Albuminuria No = 326	Albuminuria Yes = 240
	Mean or % (95% CI)	Mean or % (95% CI)
Age (Years) *	38.4 (37.0-39.8)	44.7 (43.0-46.3)
Female %	52.1 (46.7-57.5)	44.6 (38.4-51.0)
Aboriginal %	32.8 (27.9-38.1)	39.2 (33.2-45.5)
WC (CM) *	97.9 (96.1-99.8)	105.8 (103.8-107.8)
Abdominal overweight and obesity * %		
Overweight	18.5 (14.4-23.6)	19.6 (14.3-26.4)
Obesity	46.2 (40.3-52.1)	58.3 (50.7-65.6)
BMI (kg/m^2^) *	28.7 (27.9-29.6)	31.0 (30.1-31.8)
BMI % *		
25-29.9	26.6 (22.1-31.7)	22.2 (17.3-27.9)
> = 30	40.2 (35.0-45.7)	55.2 (48.8-61.4)
SBP (mmHg) *	130.0 (128.0-131.9)	137.3 (135.0-139.6)
DBP (mmHg) *	71.5 (70.1-73.0)	75.3 (73.6-77.1)
Hypertension % *	31.4 (26.6-36.7)	44.2 (38.0-50.5)
Glucose (mmol/L) *	5.5 (5.3-5.8)	6.8 (6.3-7.2)
Diabetes % *	15.0 (11.5-19.4)	35.4 (29.6-41.7)
Cholesterol (mmol/L) *	4.9 (4.8-5.0)	5.3 (5.2-5.5)
Triglycerides (mmol/L) *	1.7 (1.5-1.8)	2.4 (2.2-2.7)
HDL (mmol/L)*	1.12 (1.09-1.15)	1.08 (1.04-1.12)
Dyslipidaemia % *	42.3 (37.0-47.8)	60.0 (53.5-66.2)
GGT (IU) *	40.5 (36.0-45.0)	64.2 (53.2-75.3)
GGT > 50 % *	19.2 (15.2-23.9)	39.6 (33.4-46.1)
Smoker %	49.2 (43.8-54.7)	55.5 (49.1-61.7)
Drinking status %		
Moderate	25.6 (21.1-30.8)	20.6 (15.9-26.2)
Risky	41.5 (36.1-47.0)	44.1 (37.9-50.5)
PA sufficient %	28.2 (23.6-33.4)	26.7 (21.4-32.6)

*P < 0.05 from ttest or chi square test or corresponding non-parametric tests

Abdominal overweight was defined using WHO criteria as WC greater than 80 cm in females and 94 cm in males and obesity as greater than 88 cm in female and 102 in male

Hypertension was ascertained either by detection of high BP at examination (measured BP > =140/90 (mmHg) or previous confirmed diagnosis or currently prescribed antihypertensive medication (by medical record review)

Diabetes was defined as either clinical diagnosis verified by the participants’ medical records or a 2 hour oral glucose tolerance test, or fasting blood glucose level >7.0 mmol/L

Dyslipidaemia was defined as having triglycerides > =2.0 mmol/L or HDLC <1.0 mmol/L recommended by National Heart Foundation

As shown in Table 3, in both Indigenous populations, diabetes was strongly associated with follow-up albuminuria (OR 2.7, 95 % CI 1.8–4.2) after adjustment for age, sex, and ethnicity. Further analysis showed within the normal glucose range, that those with a baseline fasting glucose of more than 5.4 mmol/L had 2.5 times higher risk of albuminuria at follow up (OR: 2.5, 95 % CI: 1.5–4.2) compared to less than 4.7 mmol/L.

**Table 3:** Biomedical factors associated with follow-up albuminuria among Indigenous adults in 19 communities from North Queensland (OR and 95% CI)

	Aboriginal		TSI		Overall	
	Crude	Adjusted^a^	Crude	Adjusted^a^	Crude	Adjusted^b^
Abdominal overweight/obesity		“No” as reference				
Overweight	1.9 (0.8–4.3)	2.2 (0.9–5.6)	3.4 (1.2–9.9)	2.8 (0.9–8.4)	1.7 (1.0–3.0)	2.1 (1.1–3.9)
Obesity	2.3 (1.2–4.4)	5.7 (1.8–18.6)	4.2 (1.7–10.7)	8.8 (1.6–49.5)	2.0 (1.3–3.2)	5.4 (2.2–13.5)
GGT > =50 IU	“<50” as reference					
Yes	2.0 (1.1–3.6)	2.1 (1.1–4.1)	2.8 (1.6–5.1)	2.6 (1.4–5.0)	2.8 (1.9–4.1)	2.6 (1.7–3.9)
GGT tertiles	“<25 IU” as reference					
25–47	1.5 (0.7–3.4)	1.2 (0.5–2.9)	2.5 (1.4–4.3)	2.4 (1.3–4.3)	2.2 (1.4–3.3)	2.0 (1.3–3.1)
> = 48	2.5 (1.2–5.3)	2.3 (1.0–5.4)	4.2 (2.2–8.2)	4.2 (2.0–8.7)	4.0 (2.5–6.2)	3.7 (2.2–6.2)
Hypertension	“<140/90 mmHg” as reference					
Yes	2.2 (1.2–3.9)	1.6 (0.9–3.1)	1.4 (0.9–2.2)	1.0 (0.6–1.7)	1.7 (1.2–2.4)	1.2 (0.8–1.8)
Diabetes	“No” as reference					
Yes	6.1 (2.5–14.7)	5.8 (2.4–14.6)	2.5 (1.5–4.1)	2.0 (1.2–3.5)	3.1 (2.1–4.6)	2.7 (1.8–4.2)
Glucose tertiles	“<4.7 mmol//L” as reference					
4.7–5.3	1.8 (0.9–3.8)	1.7 (0.8–3.6)	1.5 (0.8–3.0)	1.2 (0.6–2.4)	1.4 (0.9–2.2)	1.4 (0.9–2.3)
> = 5.4	4.2 (2.0–9.1)	3.8 (1.7–8.4)	2.7 (1.4–5.2)	1.9 (0.9–3.9)	2.4 (1.5–3.8)	2.5 (1.5–4.2)
Current smoker	“No” as reference					
Yes	1.0 (0.6–1.8)	1.0 (0.6–1.9)	1.7 (1.1–2.7)	2.0 (1.2–3.2)	1.3 (0.9–1.8)	1.4 (1.0–2.0)

^a^adjusted for age and sex in each ethnic group, further adjusted for ethnicity in overall population

Abdominal overweight was defined using WHO criteria as WC greater than 80 cm in females and 94 cm in males and obesity as greater than 88 cm in female and 102 in male

Hypertension was ascertained either by detection of high BP at examination (measured BP > =140/90 (mmHg) or previous confirmed diagnosis or currently prescribed antihypertensive medication (by medical record review)

Diabetes was defined as either clinical diagnosis verified by the participants’ medical records or a 2 hour oral glucose tolerance test, or fasting blood glucose level >7.0 mmol/L

Dyslipidaemia was defined as having triglycerides > =2.0 mmol/L or HDLC <1.0 mmol/L recommended by National Heart Foundation

Baseline GGT > = 50 IU was associated with increased risk of albuminuria by 2.6 times (95 % CI 1.7-3.9). A dose responsive effect of GGT with albuminuria was detected. Specifically, a GGT level of 25–47 IU associated with 2-fold prevalence of albuminuria (95 % CI: 1.3–3.1) and a level of > =48 by 3.7 times (95 % CI: 2.2–6.2) compared to a level of <25 IU (p for trend <0.001). Abdominal overweight and obesity was associated with albuminuria at follow up by 2.1 (95 % CI: 1.1–3.9) and 5.4 (95 % CI: 2.2–13.5) respectively.

Aboriginal people having diabetes were 3 times more likely than TSI diabetes patients to have albuminuria at follow up. TSI smokers were twice as likely to have albuminuria compared to non-smokers, but this effect was not demonstrated among Aboriginal participants.

## Discussion

This study found a high baseline prevalence of albuminuria (20 %) among adults from rural and remote communities in north Queensland, especially among those aged over 25 years. And baseline albuminuria was associated with features of the metabolic syndrome (BMI, BP, dyslipidaemia and glucose) and higher GGT levels. This is consistent with the prevalence and associated factors reported in other Aboriginal communities in NT, SA, WA, and QLD [14–16]. The prevalence is 3 times higher than the prevalence found in the national non-Indigenous sample (AusDiab) [11].

Indigenous people in rural and remote communities had more than double the risk of follow up albuminuria at an early age compared contemporarily to the non-Indigenous Australians from the AusDiab study, similar to findings from an Aboriginal population in the NT [22]. The prevalence at follow up was more than twice of the baseline during a median 7 years of follow up.

We found consistently that increased fasting glucose or diabetes was associated with albuminuria at both occasions. The baseline prevalence of diabetes/impaired glucose tolerance in Indigenous population was between 3 and 5 times higher than the comparable rates for non-Indigenous people in all age groups from 25 years and over [23]. We have reported that the diabetes incidence in this cohort was 4 times that of non-Indigenous people and appeared to be increasing over the estimates from a decade previously [17]. As albuminuria is a key early marker of future CKD and ESRD, Indigenous Australians are at particularly high risk, and evidence for effective primary care level management is strong. Therefore early screening for albuminuria is important in this population [24]. Our finding that abdominal overweight and obesity predicted albuminuria independently of diabetes is consistent with other cohort studies among south Asians and Chinese [25, 26]. Apart from secondary prevention of ESRD with drugs, surgical intervention to reduce obesity has proved highly effective in reducing albuminuria incidence among obese Swedish subjects [27]. The underlying mechanisms may include renin-angiotensin-aldosterone system activation, leptin, adiponectin, fetuin-A, and adipose tissue inflammation, as factors that contribute to glomerulonephritis, focal and segmental glomerulosclerosis, and IgA nephropathy [28].

Tobacco smoking is highly prevalent among Indigenous Australians and was associated with albuminuria at both baseline and follow up. This finding is similar to other studies in Europeans, Americans and Asians [29–31]. Smoking predicted albuminuria or proteinuria in the Framingham Offspring Cohort [32] and Japanese adults [33] but not among the European PREVEND cohort [34].

GGT, as a marker of liver disease and alcohol intake and as a biomarker of oxidative stress [35], was associated with albuminuria in a dose-responsive manner in the two Indigenous populations after adjustment for demographics, overweight and obesity, diabetes, hypertension, and smoking and drinking habits. The results were similar to the systematic review on the association between GGT and albuminuria or CKD in both cross sectional and follow up studies among Americans and Koreans [36]. We have reported that GGT predicts hypertension incidence [37] and is highly correlated with overweight and obesity. Metabolic syndrome and alcohol drinking magnifies the association between GGT and metabolic components among this population [38, 39]. Further exploration of its association with cardiovascular conditions and diabetes is needed to understand the additional independent impact of GGT on albuminuria found in this population for better risk evaluation or stratification in Indigenous population.

Strengths of this study include a representative community-based sample of both Aboriginal and TSI populations and objective clinical measurements. TSIs are the second group of Indigenous Australians who inhabit the islands of Torres Strait as a part of Queensland. They have a heavier phenotype and a healthier lifestyle compared to Aborigines. The data collected in this study enabled us to compare albuminuria and its associated biomedical factors between the two ethnic groups. Although the risk profiles in the two populations are different, the albuminuria prevalence was not statistically different, partly due to the higher risk for Aboriginal people given the same level of increased glucose or diabetes, and/or less risk for Aboriginal smokers compared to TSIs, or disproportionally increased loss of Aboriginal participants, including those with baseline albuminuria.

Limitations should be noted. Firstly, the definition of albuminuria was based on a single UACR measurement due to the study design of screening a large general population from communities with limited resources, which could misclassify cases. The follow up data showed a small number of baseline micro albuminuria cases were normal at follow up. Secondly, the follow up information was not completed to sufficiently facilitate the estimation of the incidence rate, especially for those who moved from the communities. Thirdly, a large number of relatively healthier Aboriginal participants lost contact at the follow up which could result in overestimate of the prevalence at follow up. Lastly, we could not detail medical and family history, and some potential confounders to explore albuminuria and its predictors. For example, studies among Aboriginal Australians in remote communities show that earlier infection is strongly associated with kidney dysfunction in later life [40, 41]. In spite of these, this study documented the prevalence from a representative population for both Aboriginal and TSI groups in rural and remote communities in North Queensland at baseline and the follow up albuminuria in a selected group of high risk participants including important biomedical factors.

## Conclusions

The high prevalence of albuminuria in the Indigenous Australian population was associated with excessive risk of glycaemia, diabetes, obesity, increased GGT, or smoking. Early screening and management of these factors are essential to stem the tide of ESRD and CVD in this population.

## Additional files

Additional file 1:
**Table S1.** Characteristics of participants by follow up status. (DOCX 20 kb) Additional file 2:
**Table S2.** Characteristics of participants at follow up by ethnicity. (DOCX 21 kb)

## Abbreviations

CKD: Chronic kidney disease
AusDiab study: The Australian Diabetes, Obesity and lifestyle Study
UACR: Urine albumin creatinine ratio
TSI: Torres strait islanders
NT: Northern Territory
SA: South Australia
WA: Western Australia
QLD: Queensland
ESKD: End-stage kidney disease
BP: Blood pressure
PA: Physical activity
GGT: Gamma-glutamyl transferase
HDLC: High density lipoprotein cholesterol
WC: Waist circumference
OR: hazard ratios
