# Supplementary material for: Albuminuria and its associated biomedical factors among indigenous adults in Far North Queensland: a 7-year follow up study
Source: BMC Nephrol. 2015 Dec 10;16:208. doi: 10.1186/s12882-015-0200-8 (PMC4676181; doi:10.1186/s12882-015-0200-8)
Supplement: Additional file 1: — Table S1. Characteristics of participants by follow up status. (DOCX 20 kb) [file 12882_2015_200_MOESM1_ESM.docx]

**Table S1. Characteristics of participants by follow up status**

|  | **Dead n=182** | **Prisoned n=21** | **Moved away n=427** | **Unknown n=775** | **Completed n=566** |
| --- | --- | --- | --- | --- | --- |
|  | **Mean or % (95% CI)** | **Mean or % (95% CI)** | **Mean or % (95% CI)** | **Mean or % (95% CI)** | **Mean or % (95% CI)** |
| **Age (Years) *** | 53.7 (51.3-56.1) | 27.3 (24.2-30.3) | 34.4 (33.1-35.7) | 36.1 (35.2-37.1) | 41.1 (40.0-42.2) |
| **Female %** | 47.3 (40.1-54.5) | 4.8 (0.6-29.2) | 50.8 (46.1-55.5) | 52.9 (49.4-56.4) | 48.9 (44.8-53.1) |
| **Aboriginal % *** | 62.6 (55.4-69.4) | 81.0 (58.2-92.8) | 49.4 (44.7-54.2) | 61.9 (58.5-65.3) | 35.5 (31.7-39.6) |
| **WC (CM) *** | 96.7 (94.4-99.0) | 90.9 (84.9-96.9) | 96.2 (94.6-97.7) | 95.8 (94.7-97.0) | 101.3 (99.9-102.7) |
| **Abdominal overweight and obesity * %** | | |  |  |  |
| Overweight | 17.9 (12.5-25.1) | 17.6 (5.6-43.7) | 18.3 (14.6-22.6) | 20.8 (17.9-24.1) 9) | 19.0 (15.6-22. |
| Obesity | 43.4 (35.6-51.7) | 5.9 (0.8-33.4) | 41.3 (36.3-46.5) | 43.0 (39.3-46.8) 4) | 50.8 (46.1-55. |
| **BMI (kg/m^2^) *** | 26.7 (25.7-27.7) | 25.4 (22.8-28.0) | 28.6 (28.0-29.2) | 27.6 (27.1-28.1) | 29.7 (29.1-30.3) |
| **BMI % *** |  |  |  |  |  |
| 25-29.9 | 25.3 (19.5-32.1) | 28.6 (13.2-51.4) | 27.5 (23.5-32.0) | 27.9 (24.9-31.2) | 24.7 (21.3-28.5) |
| >=30 | 33.0 (26.5-40.1) | 23.8 (10.0-46.7) | 38.1 (33.6-42.8) | 32.3 (29.1-35.7) | 46.6 (42.5-50.8) |
| **SBP (mmHg) *** | 139.7 (136.0-143.4) | 126.7 (121.8-131.5) | 129.0 (127.3-130.7) | 129.9 (128.6-131.2) | 133.1 (131.5-134.6) |
| **DBP (mmHg) *** | 75.9 (73.8-78.1) | 63.7 (59.3-68.0) | 70.5 (69.3-71.8) | 72.0 (71.1-72.9) | 73.2 (72.0-74.3) |
| **Hypertension % *** | 49.2 (41.9-56.4) | 14.3 (5.4-36.9) | 25.6 (21.7-30.0) | 26.0 (23.0-29.2) | 36.8 (32.9-40.9) |
| **Glucose (mmol/L) *** | 6.7 (6.2-7.2) | 4.8 (4.4-5.3) | 5.5 (5.3-5.8) | 5.7 (5.5-5.9) | 6.1 (5.8-6.3) |
| **Diabetes % *** | 28.6 (22.5-35.6) | 4.8 (0.6-28.2) | 11.0 (8.4-14.4) | 12.8 (10.6-15.3) | 23.7 (20.3-27.4) |
| **Cholesterol (mmol/L) *** | 5.0 (4.9-5.2) | 4.7 (4.2-5.1) | 5.0 (4.9-5.1) | 4.9 (4.8-5.0) | 5.1 (5.0-5.2) |
| **Triglycerides (mmol/L) *** | 2.0 (1.8-2.3) | 2.3 (1.3-3.4) | 1.8 (1.6-1.9) | 1.9 (1.8-2.0) | 2.0 (1.8-2.1) |
| **HDL (mmol/L)** | 1.13 (1.08-1.18) | 1.02 (0.91-1.13) | 1.15 (1.12-1.18) | 1.15 (1.13-1.17) | 1.10 (1.08-1.13) |
| **Dyslipidaemia %** | 54.2 (46.8-61.4) | 57.9 (35.0-77.8) | 43.3 (38.6-48.2) | 45.8 (42.3-49.4) | 49.7 (45.5 -53.9) |
| **GGT (IU) *** | 65.9 (52.1-79.8) | 77.5 (44.3-110.7) | 43.7 (39.2-48.2) | 52.8 (48.5-57.1) | 50.4 (45.1-55.8) |
| **GGT>50 % *** | 39.1 (32.2-46.5) | 47.4 (26.3-69.4) | 25.8 (21.8-30.3) | 32.8 (29.5-36.2) | 27.7 (24.1-31.6) |
| **Smoker % *** | 52.2 (44.9-59.4) | 80.0 (56.5-92.5) | 58.5 (53.8-63.1) | 63.5 (60.0-66.9) | 51.9 (47.7-56.0) |
| **Drinker %** |  |  |  |  |  |
| Moderate | 16.8 (11.9-23.1) | 20.0 (7.5-43.5) | 27.4 (23.4 (31.9) | 21.2 (18.4-24.3) | 23.5 (20.1-27.2) |
| Risky | 39.3 (32.3-46.8) | 75.0 (51.5-89.5) | 49.6 (44.9-54.4) | 54.4 (50.8-57.9) | 42.6 (38.5-46.8) |
| **PA sufficient %** | 18.1 (13.2-24.4) | 28.6 (13.2-51.4) | 26.0 (22.0-30.4) | 27.6 (24.0-31.4) | 22.2 (19.4-25.3) |

*P<0.05 from oneway ANOVA or chi square test or corresponding non-parametric tests;

Abdominal overweight was defined using WHO criteria as WC greater than 80 cm in females and 94 cm in males and obesity as greater than 88 cm in female and 102 in male;

Hypertension was ascertained either by detection of high BP at examination (measured BP >=140/90 (mmHg) or previous confirmed diagnosis or currently prescribed antihypertensive medication (by medical record review);

Diabetes was defined as either clinical diagnosis verified by the participants’ medical records or a 2 hour oral glucose tolerance test, or fasting blood glucose level >7.0 mmol/L;

Dyslipidaemia was defined as having triglycerides >=2.0 mmol/L or HDLC <1.0 mmol/L recommended by National Heart Foundation;
